# Pancreatic cancer derived 3D organoids as a clinical tool to evaluate the treatment response

**DOI:** 10.3389/fonc.2022.1072774

**Published:** 2023-01-12

**Authors:** Hem D Shukla, Tijana Dukic, Sanjit Roy, Binny Bhandary, Andrew Gerry, Yannick Poirier, Narottam Lamichhane, Jason Molitoris, France Carrier, Aditi Banerjee, William F. Regine, Jerimy C. Polf

**Affiliations:** ^1^ Division of Translational Radiation Sciences, Department of Radiation Oncology, University of Maryland, School of Medicine, Baltimore, MD, United States; ^2^ Division of Medical Physics, Department of Radiation Oncology, University of Maryland, School of Medicine, Baltimore, MD, United States; ^3^ Department of Pediatrics, University of Maryland School of Medicine, Baltimore, MD, United States

**Keywords:** pancreatic cancer, 3D organoids, chemotherapy, chemo-radiation therapy, radiation, treatment response

## Abstract

**Background and purpose:**

Pancreatic cancer (PC) is the fourth leading cause of cancer death in both men and women. The standard of care for patients with locally advanced PC of chemotherapy, stereotactic radiotherapy (RT), or chemo-radiation-therapy has shown highly variable and limited success rates. However, three-dimensional (3D) Pancreatic tumor organoids (PTOs) have shown promise to study tumor response to drugs, and emerging treatments under *in vitro* conditions. We investigated the potential for using 3D organoids to evaluate the precise radiation and drug dose responses of *in vivo* PC tumors.

**Methods:**

PTOs were created from mouse pancreatic tumor tissues, and their microenvironment was compared to that of *in vivo* tumors using immunohistochemical and immunofluorescence staining. The organoids and *in vivo* PC tumors were treated with fractionated X-ray RT, 3-bromopyruvate (3BP) anti-tumor drug, and combination of 3BP + fractionated RT.

**Results:**

Pancreatic tumor organoids (PTOs) exhibited a similar fibrotic microenvironment and molecular response (as seen by apoptosis biomarker expression) as *in vivo* tumors. Untreated tumor organoids and *in vivo* tumor both exhibited proliferative growth of 6 folds the original size after 10 days, whereas no growth was seen for organoids and *in vivo* tumors treated with 8 (Gray) Gy of fractionated RT. Tumor organoids showed reduced growth rates of 3.2x and 1.8x when treated with 4 and 6 Gy fractionated RT, respectively. Interestingly, combination of 100 µM of 3BP + 4 Gy of RT showed pronounced growth inhibition as compared to 3-BP alone or 4 Gy of radiation alone. Further, positive identification of SOX2, SOX10 and TGFβ indicated presence of cancer stem cells in tumor organoids which might have some role in resistance to therapies in pancreatic cancer.

**Conclusions:**

PTOs produced a similar microenvironment and exhibited similar growth characteristics as *in vivo* tumors following treatment, indicating their potential for predicting *in vivo* tumor sensitivity and response to RT and combined chemo-RT treatments.

## Introduction

Pancreatic cancer is the fourth most lethal cancer in the United States. The current standard of care for resected patients consists of stereotactic radiation therapy (RT), chemotherapy or chemo-RT, and despite extensive research and development of new treatment regimens, 5-year survival remains at < 8%. Pancreatic cancer patients receiving RT, chemotherapy or chemo-RT are most commonly prescribed a regimen based on guidelines for similar tumors with similar histology and stage, as published by the National Comprehensive Cancer Network (1–4). These guidelines are based on large-scale phase III clinical trials used to study and quantify overall treatment response and outcomes for the entire population of patients with similar tumor characteristics, with a limited role for incorporating genetic profiling or molecular information. Variations in tumor and normal tissue response to radiation have long been known (5–8) with considerable progress over the last 10–15 years in understanding clinical manifestations and biologic mechanisms of both tumor response and normal tissue toxicities (acute and late).

Several factors influence and complicate a patient’s likelihood of tumor response to RT or development of associated normal tissue toxicities. These complex factors are related to physics (total dose, dose per fraction and volume irradiated, and dose homogeneity), combined with use of concomitant chemotherapy and/or surgery, patient characteristics (age, environmental factors, hemoglobin levels, and comorbid conditions) (9, 10). Furthermore, variations in patient sensitivity to RT and combined chemo-RT have been investigated, with the first report in 1975 of fibroblasts from one patient being 3× more sensitive than those from a cohort of normal donors (11). Several subsequent studies showed differential *in vitro* radiosensitivity in primary cancer cell culture (12–16). This has led to a large amount of research into development of biomarkers and models that can predict sensitivity of individual patients’ tumors to RT, chemotherapy, and chemo-RT, including standard 2D *in vitro* cell survival studies and 3D xenograft models (17– 18).

In addition, the study and use of 3D patient-derived tumor organoid models to study tumor response to existing and emerging treatment regimens have grown substantially in recent years (19). Tumor Derived Organoids delineate more accurate response than cancer cell lines under 2D conditions in terms of reiterating the *in vivo* environment, and amenable for easier manipulation and high throughput screening than xenografts model that takes longer time to achieve. Thus, development of precision medicine approach customized to individual patients could be more promising by adapting to an appropriate platform such as bioengineered matrix that has the ability to reproduce patient responses to treatment (20). There has also been development of floating spheroid culture model that allowed 3-D culture of cancer cells, however this organoid system lacked matrix attachment and showed variable growth pattern (21). Matrigel based 3D culture system is routinely used for organoid growth and manipulation, however, it lacks defined composition and exhibits batch to batch variation (22). Furthermore, hydrogels like polyethylene glycol which are synthetic polymers, offer excellent elasticity in the control of their physicochemical properties (23), however, these hydrogels are less biocompatible than hydrogels based on natural materials. Recently 3D collagen-based scaffold culture system has also been used with zebrafish model combined with the use of patient-derived primary cultures to examine trabectedin response in undifferentiated pleomorphic sarcoma which has shown clinical promise (24). In recent report, collagen-based scaffolds model has been successfully established to study pathophysiological features of oropharyngeal squamous cell carcinoma and it has been used to understand drug-resistance processes (25). These developments have been driven by the limited efficacy in translating results derived from 2D *in vitro* cell cultures into positive clinical outcomes (26, 27). While benefiting from simplicity and relative ease of use, 2D cultures lack the ability to reproduce the diversity of cellular organization, extracellular microenvironment, and cellular contact and signaling pathways found within the tumor (28–32). Patient-derived organoid models have shown the ability to accurately model *in vivo* tumors, reproducing the heterogeneous tissue and cellular microenvironments while maintaining genetic and phenotypic profiles of the original tumors from which they were derived (27).The majority of 3D organoid studies have focused primarily on tumor response to chemotherapy and other targeted treatment agents (19, 33). A few studies have investigated RT and chemo-RT responses in 3D tumor organoids derived from glioblastoma (34), head and neck (35, 36), and rectal/colorectal (37–39) tumors.

Although several studies have outlined the development and use of pancreatic tumor organoids (PTO) derived from mouse or human cells (28, 29, 40–42) and these reports have mainly focused on the general response of PTO organoids and individual patient sensitivity to current and emerging chemotherapy, molecular, and immunotherapy methods. To date, few studies have been published on the applicability of 3D organoids for modeling the response of pancreatic cancer to RT and combined chemo-RT. Here we report on an initial feasibility study of PTO organoids and their ability to accurately reproduce the responses to fractionated RT and chemo-RT of the *in vivo* tumors from which they were derived. PTO organoids were created from mouse pancreatic tumors, and the microenvironments of the PTO organoids were compared to excised *in vivo* tumor tissues. Responses of the PTO organoids were compared to the responses of *in vivo* tumors to treatment with fractionated x-ray RT. In addition, to illustrate the potential of PTO organoids to determine tumor sensitivity to multiple treatments, the responses of the PTO organoids to 3-bromopyruvate (3BP) and combined 3BP-RT treatments were

studied and compared PTO responses to RT alone.

## Materials and methods

### Subcutaneous tumor xenografts

After animal protocol approval from our institution’s Office of Animal Welfare Assurance (IACUC Protocol #1019006), 6–7-week-old C57Bl/6 mice were subcutaneously implanted with pancreatic tumors derived from Panc2 cells in the right flank. A full description of the subcutaneous tumor generation can be found in the Supplemental Materials. This included 20 mice in which to study the growth of untreated (control; *n* = 10) and RT-treated (*n* = 10) *in vivo* tumors. Tumor tissues from untreated control animals were used to produce PTO organoids.

### Generation and propagation of mouse tumor derived organoids

The full procedure for organoid production can be found in the Supplemental Materials. In brief, animals were euthanized with CO_2_ when the tumor size reached 1.2–1.5 cm. Tumors were excised and washed with cold DMEM/F12 medium and processed for organoid production as described earlier (33, 43). The finely minced tumor tissues were digested in Tissue Digestion Cocktail (Collagenase IV (3.75 ml), Dispase (3.75 ml) and DNase I (1 mg/ml) in DMEM/F12 (22.2 ml) with 15 mM HEPES) for 20 min. The digested tissue fragments were allowed to settle by gravity for 5 min and supernatant was carefully removed and passed through 70 µm strainer. The pass through was discarded and tissues and ductal fragments were collected in 10 ml chilled DMEM/F12 in 15 ml sterile falcon tube. Subsequently, tubes were centrifuged at 300 X g for 5 min. Pellet was suspended into 25 µl chilled Matrigel and mixed 5-8 times with cold pipette tip and cultured as dome in prewarmed 24 well plate. The plates with Matrigel domes were then incubated at 37°C for 10 minutes with 5% CO2, and 0.5 mL pancreatic cult media containing 2.5 ng/ml recombinant EGF, 2.5 ng/ml FGF, (R&D Systems), 2% v/v vitamin B27, 200 μg/ml ALK5 inhibitor, 0.021 mg/ml gastrin, 81.5 mg/ml N-acetylcysteine, 122 mg/ml Nicotinamide, 1:1000 Y-27632 (Rho-kinase inhibitor) (Sigma) in 100 ml DMEM/F12 GlutaMAX (ThermoFisher Scientific, Waltham, MA). For coculturing, t cancer associated fibroblasts (CAF) were isolated from tumor tissues and cultured in 60 mm cell culture dish in DMEM/F12 media with 5% FBS. After completion of the three passaging CAF were mixed with organoids in 1:4 ratio and cultured in Matrigel dome in DMEM/F12 minimal media lacking noggin, TGF-β inhibitor and vitamin B27.

The organoid culture was maintained in the incubator for 3-4 days to allow organoids to grow. On day four the plates were treated, and organoid growth was monitored using an EVOS cell imaging system (ThermoFisher Scientific; Waltham, MA) at 4x magnification under bright-field light condition for 10 days following treatment.

### *In vivo* radiation treatment

*In vivo* tumors on mouse flanks were irradiated at ~2.5 Gy/min using the Small Animal Radiation Research Platform (SARRP; Xstrahl; Suwanee, GA) operating at 220 kVp, 0.15-mm Cu filtration (0.6-mm Cu half-value layer), and 13 mA. Mice were irradiated sequentially, placed in groups of 3 on the SARRP’s robotic stage using a bilateral anterior–posterior/posterior–anterior (AP/PA) treatment geometry with the standard 10-mm collimation at a source-to-axis distance of 35 cm. The stage was first translated from one animal to the next to deliver the AP treatment field, then the gantry was rotated from 0° to 180° and the PA field delivered to each mouse. Irradiation times were calculated using CT scans from representative animals in the SARRP treatment planning system and verified using a mouse-mimicking plastic phantom and a pinpoint ionization chamber (IBA; Louvain-La-Neuve, Belgium) cross-calibrated with the National Institute of Standards and Technology (NIST)–traceable Farmer chamber.

Twenty mice (n = 20) with flank tumors were divided into two treatment groups with ten mice (N = 10) per group: 1) control (no treatment), and 2) 8 Gy RT. The *in vivo* tumors on the mice flanks were treated with fractionated X-ray RT to a total dose of 8 Gy. The RT was delivered in 2 Gy fractions on subsequent days.

### Treatment of organoids with anti-tumor drugs and RT

For the RT treatment delivery, the co-cultured organoids were irradiated at 2.3 Gy/min using an Xrad 320 biological irradiator (Precision X-Ray; Madison, CT) operating at 320 kVp, 2-mm Al filtration, and 13 mA (1-mm Cu half-value layer). Samples were placed at 50-cm source-to-surface distance on a foam surface to eliminate backscatter. Organoids were irradiated in an AP direction, creating geometric conditions closely matching those used for reference dosimetry. Irradiators for both *in vivo* tumor and organoids irradiations were calibrated using a NIST-traceable PTW TN30013 Farmer-type ionization chamber and a PTW Unidose electrometer (PTW Freiburg; Breisgau, Germany) following the American Association of Physicists in Medicine (AAPM) Task Group 61 in-air calibration protocol for X-ray irradiators (44).

To evaluate proliferation and response of organoids to fractionated RT, organoids were treated with doses of either 4, 6 or 8 Gy. Irradiation treatments were delivered in fractions of 2 Gy/fraction, with one fraction delivered per day on subsequent days until the entire dose was delivered. Moreover, to study effect of drug 3BP treatment, organoids were treated with 100 µM concentration (IC50) for 24 hours and growth response was monitored and recorded by bright field imaging. In addition, to study the combinatorial dose response of 3BP with RT, organoids were first treated with 100 µM of 3BP for 24 hours, followed by 4 Gy RT (delivered in two fractions as described above).

After all treatments, the media was changed, and organoids were allowed to grow for 10 days. For each treatment, five (n=5) organoids were studied. RT and drug induced changes in organoids morphology was recorded once a day (every 24 hours) for 10 days after chemo treatment or delivery of the final fraction of RT treatment using brightfield imaging obtained at 4× and 10× magnification with an EVOS microscope (ThermoFisher Scientific, Waltham, MA).

### Hematoxylin and eosin staining

Hematoxylin and eosin (H&E) staining of treated and untreated organoids was performed after organoids were fixed with 10% formalin for 24 hours at room temperature and washed with milliQ water before treatment with Vector Hematoxylin QS (H-3404-100; Vector Laboratories; Burlingame, CA) and Eosin Y (HT110116; MilliporeSigma; Burlington, MA). Organoids with Matrigel were rinsed with ethanol and then MilliQ water between each staining phase. Images were captured with EVOS microscopy at 4× and 10× magnification under bright field.

### Immunofluorescence and imaging

Organoids were grown in 24 well plates in 50% Matrigel (untreated or treated as described above) were fixed in 10% formalin for 12 hours, washed with 1× PBS for three times, and treated with 0.2% triton x-100 in 1× PBS for 5 minutes. The triton x-100 was removed and incubated with blocking reagent (5% goat serum, 1% bovine serum albumin) in 1× PBS for 1 hour. The blocking reagent was removed, and the tumor tissues and organoids were incubated with either: 1) a primary antibody anti-mouse vimentin (Biolegend, USA) in a 1:100 dilution, or 2) with primary antibody anti-rabbit cleaved caspase-3 (Cell Signaling Technology; Danvers, MA) in a 1:300 dilution in a cold room overnight. The next day, the antibody was removed and washed with 1× twin-buffered saline tween (TBST) for 5 minutes each for three times. Tumor tissues and organoids were incubated with fluorescently labeled secondary antibody Alexa fluor 488 (ThermoFisher Scientific; Waltham, MA) in a 1:400 dilution. After washing with 1× TBST, incubated with Hoechst dye 1 μg/mL for 15 minutes and washed with 1× PBS for three times. Finally, 1× PBS was added to the labeled organoids, and fluorescence images were acquired with green and 4′,6-diamidino-2-phenylindole (DAPI) acquisition fields.

### Co-staining of pancreatic tumor organoids with αSMA and vimentin antibodies

The pancreatic tumor organoids were passaged as described previously. The immunofluorescence staining was performed as follows; The organoids were fixed in tissue fixation buffer containing formalin solution, 10% neutral buffer overnight (Sigma Chemical, USA). The following day, the fixing was stopped, and organoids were washed with 1 X PBS 3 times for 5 minutes each. Subsequently, organoids were treated with 0.2% Triton X-100 followed by blocking with 5% goat serum with 1% BSA for 1 hour. We used α SMA primary antibodies, rabbit polyclonal unconjugated at 1:100 dilution (ABclonal, USA) and Alexa fluor 594 conjugated anti-Vimentin (mouse) at 1:300 dilution for overnight in 1:5 dilution in blocking buffer. After completion of the incubation, primary antibodies were removed, and organoids were washed as described above. In addition, Alexa fluor 488 rabbit secondary antibody (ThermoFisher Scientific, USA) was used at 1:300 dilution for 1 hour to detect unconjugated αSMA. Images were captured using EVOS Microscope (ThermoFisher Scientific, USA) under dark condition.

### Analysis of tumors and tumor organoids response to drugs and radiation treatment

**Figure 1:**
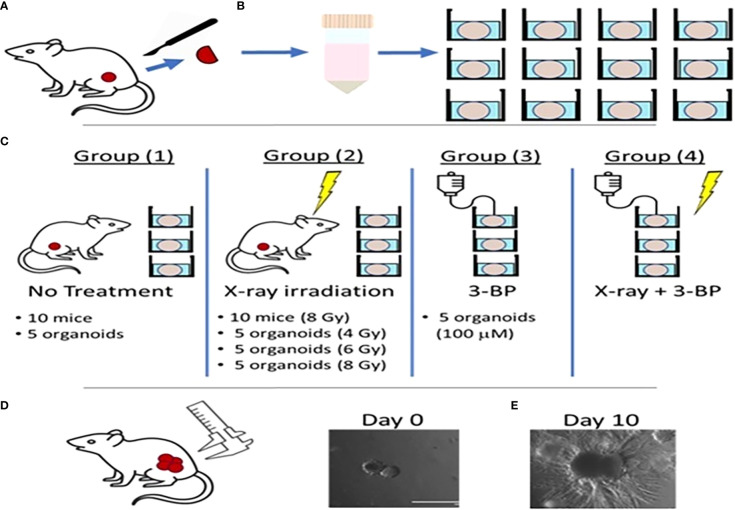
Schematic diagram showing radiation and drug treatment in tumor organoids.**(A)** production of tumor organoids from *in vivo* tumor. **(B)** Tumor tissues resected from mouse flanks were digested and used to grow 3D PDAC organoids. **(C)** Mice with flank tumors were randomized to either control or 8-Gy irradiation groups, and 3D organoids were randomized to control, irradiation only (4, 6,or 8 Gy), 3BP only, or 4 Gy irradiation + 3BP treatment groups. Treatment response was determined by measuring the size of **(D)** *in vivo* flank tumors with calipers and **(E)** organoids using bright-field microscopy imaging for 10 days following treatment.

Tumor tissues from 8 untreated control animals were resected and digested and processed for organoid culture, we observed growth in about 24 hours and passaged after four days. Once organoids reached 100 μm in size they were treated as follows (**Figure 1**) (1): irradiation with different doses of (4, or 8 Gy) RT (2); 100 µM of 3BP; and 3) combined 4 Gy RT and 100 µM 3BP anti-cancer drug. Untreated organoids were considered as control. Five (n=5) organoids for each group were imaged using an EVOS imaging system every day for 10 days starting after chemo or the final RT treatment fraction. The size of the organoids was determined as the area (A = length × width) of a rectangular box placed around the edges of the organoids (**Supplemental Material, Figure S2**). The average growth rate factor of organoids was calculated as size on a specified day (N) divided by the size on day 0, for untreated (control), and for treatment response, the volume of treated organoids was recorded as 0 day at first day of treatment, and after 10 days of treatment it was recorded as N and the relative growth was calculated as g= (N/0).

For *in vivo* experiment, twenty mice with tumors were randomized into two treatment groups of ten (n=10) mice each (1): no treatment (controls); and (2) fractionated RT to a total dose of 8 Gy (2 Gy/fraction, 1 fraction/per day x 4 days). Following treatment, the sizes of *in vivo* tumors were measured every day for 10 days. Tumor size was determined in terms of volume using the formula: V = [W^2^ x L]/2, where V is tumor volume, W is tumor width, and L is tumor length, each measured using electronic calipers (45).

### Statistical analyses

The response of the organoids to each type of treatment was determined from the difference in growth of the control organoids and the organoids subjected to a given type of treatment. Since each comparison involved only two data groups [ (1) control and (2)] individual treatment type], the statistical significance of the difference between the average size of untreated control organoids and individual treatment type organoids was calculated using an unpaired one-sided student t-test (*P* value) (46). Differences in average organoid size were considered significant for *P* < 0.05 and considered highly

significant for *P* < 0.01.

## Results

### Growth and molecular characterization of pancreatic tumor organoids

**Figure 2:**
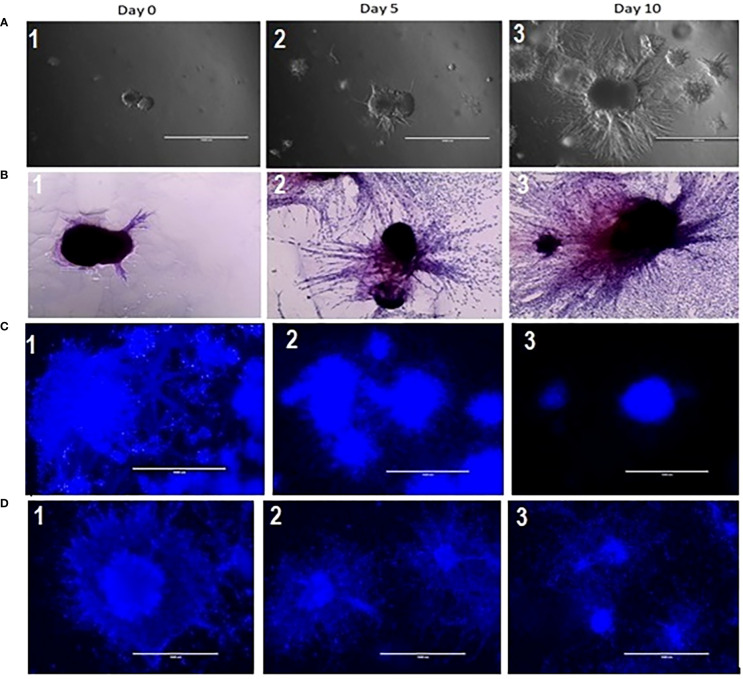
**(A)** Growth characterization of pancreatic tumor organoids. Top: Bright-field images of organoids on days 0, 5, and 10; 2 **(B)**: H&E staining of tumor organoids on days 0, 5, and 10. Scale bar = 1000 µm; **Figure 2** **(C)** (2&3): Growth inhibition in tumor organoids treated with 10 and 100 µM of 5 FU as compared to control (2C1); 2 **(D)**: (2 &3) Growth inhibition in tumor organoids treated in combination of 10 & 100 µM of 5 FU with 8 Gy RT as compared to control (2D-1).

To establish and characterize the preclinical PDAC organoid model, we first performed an initial evaluation of the shape and growth of the untreated control organoids. As shown in **Figure 2**, PDAC organoids initially developed as dense spheroids and grew into an asymmetric and moderately loose aggregate spheroid over 10 days (**Figure 2A**,1-3). In the untreated control samples, fibrotic outgrowth of the PDAC organoids could be seen as early within 24 hours of incubation at 37°C, with a rapid, fibrotic cellular outgrowth over the 10 days of observation. The H & E staining of the organoids further illustrates the extent of the aggressive fibrotic outgrowth of pancreatic tumor organoids (**Figure 2B**, 1-3). The full extent of the growth of the organoids’ dense central cores as well as the growth of the fibrotic arms (combining with outward cellular growth of other organoids) were found in day 5 and 10. This is especially apparent in the H&E-stained organoids, where they exhibited aggressive growth and proliferation of the dense cores. In addition, tumor organoids showed significant growth inhibition when treated with 5-FU (10 and 100 µM) alone (**Figure 2C**, 1-3), or in combination with 6 Gy of RT (**Figure 2D**, 1-3).

**Figure 3:**
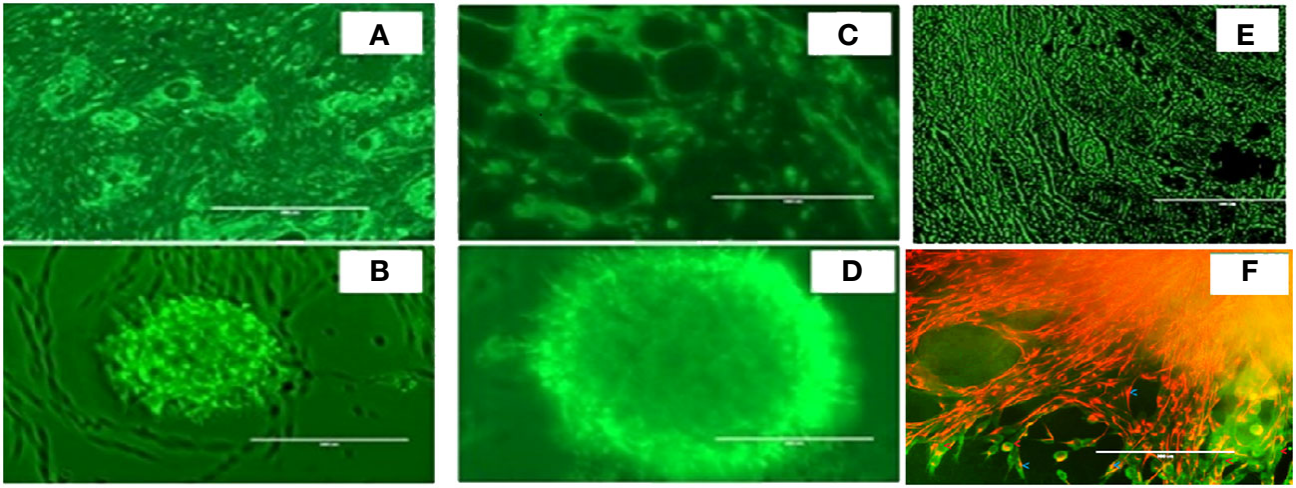
Expression of α-SMA, TGFβ and Vimentin markers in mouse pancreatic tumor tissues and tumor derived organoids. Immunofluorescence images of (top row) *in vivo* tumor tissues, and (bottom row) organoids indicating the expression of the biomarkers **(A, B)** TGF-β, **(C, D)** a-SMA, **(E, F)** vimentin costaining in red, and αSMA in green indicating the presence of cancer associated fibroblasts in both the organoids and the *in vivo* tumors they were derived from. Scale bar represents 200 µm.

As shown in **Figure 3**, we again observed that both *in vivo* tumor tissues and organoids displayed the strong fibrotic growth features that is characteristic of pancreatic cancer similar to that seen in **Figure 2** showing similar aggressive growth in tissues and 3D growing organoids. It was noticeable that tumor tissues and tumor derived organoids shared the same microenvironment, and IHC images showed expression of TGF-β (**Figures 3A, B**), and α-SMA (**Figures 3C, D**) as their expression was monitored using immunofluorescence. Moreover, we also observed expression of vimentin in tumor tissues and organoids indicates the presence of cancer associated fibroblasts (**Figures 3E, F**). The similar observation was revealed when PDAC organoids were co-cultured with tumor fibroblast and after co-staining presence of vimentin in CAF (red stain) and αSMA was identified in cancer associated fibroblasts (**Figure 3F**; **Supplementary Figure 8**). However, the PDAC organoids alone, showed absence of fluorescence signal suggesting absence of fibroblast cells (**Supplementary Figure S1**) in the organoids. This indicates the importance of cellular components in the pancreatic tumor microenvironment which have been shown to affect PDAC resistance to radiation therapy.

**Figure 4:**
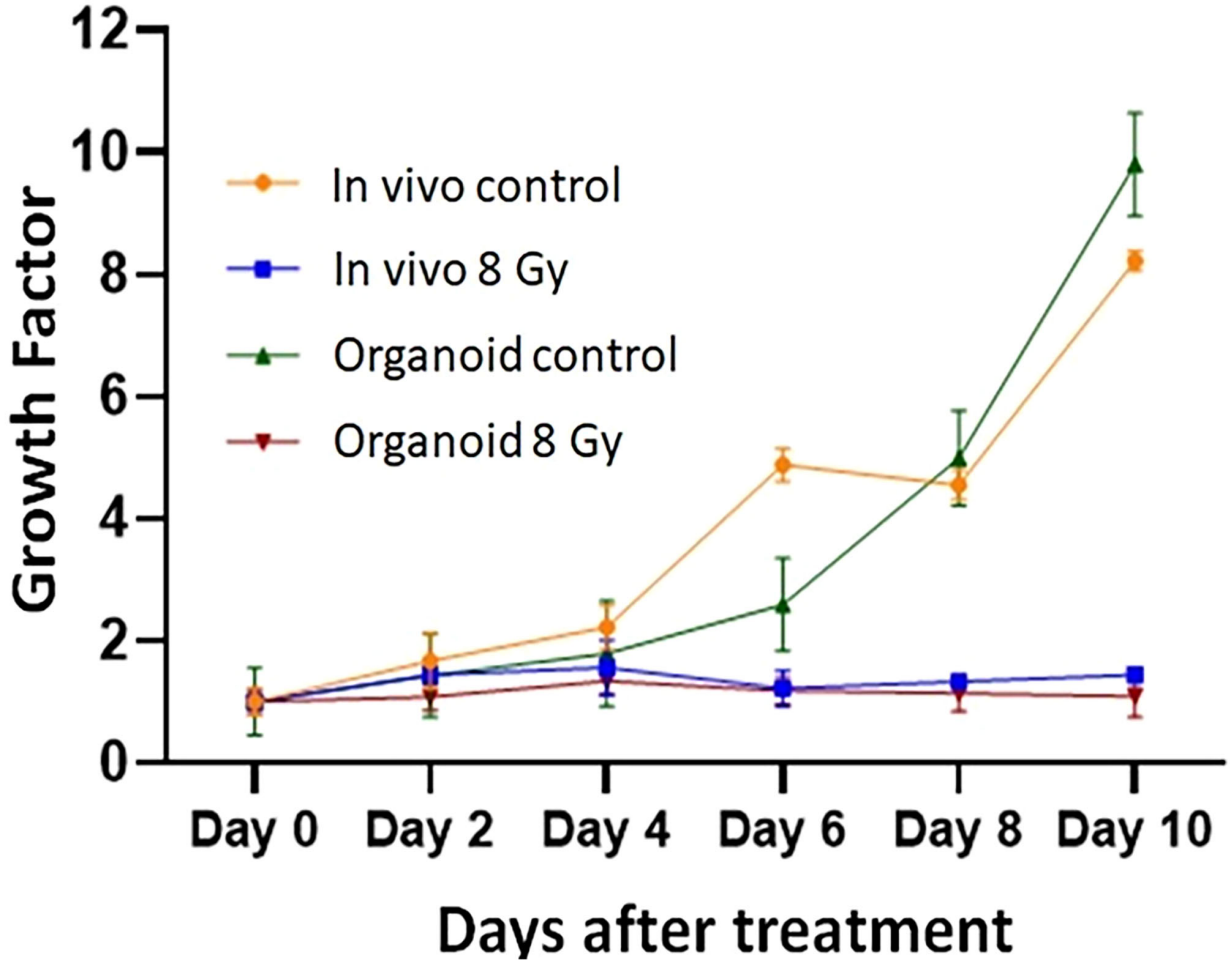
Comparison of radiation treatment response *in vivo* tumor, and tumor derived organoids. Data points represent average of all samples for a given treatment group, and error bars are 1s standard deviation of sample values.

**Figure 5:**
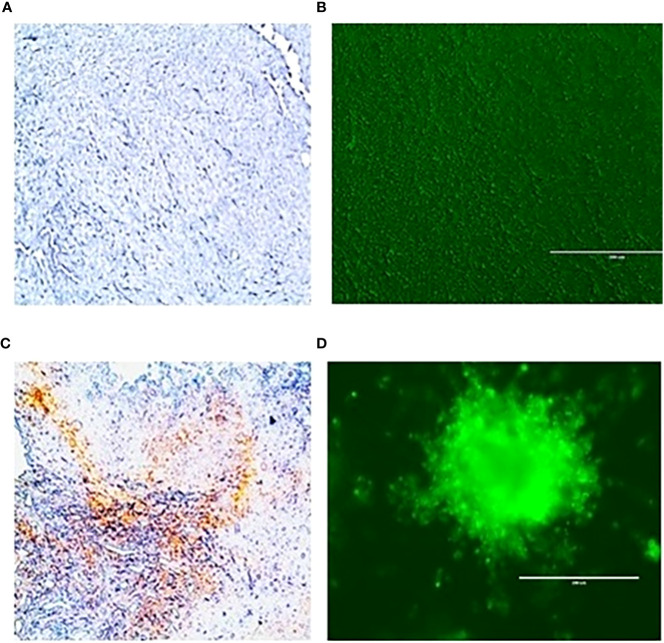
Expression of caspase 3 an apoptotic marker *in vivo* tumor tissues and organoids. **(A)**, untreated control and after treating with 100 µM 3-BP **(C)**; Tumor organoids untreated **(B)**, and after treating with 100 µM 3-BP antitumor drug **(D)**.

To evaluate the radiation response in tumor organoids proliferation and *in vivo* tumor growth, organoids and tumor were treated with 8 Gy radiation in two fractions. The results indicated the tumor-derived organoids and *in vivo* tumors exhibited similar growth rates over the 10 days. Whereas growth inhibition in both organoids and *in vivo* tumors was observed in the treated group after day four, and inhibition was consistent (**Figure 4**). The mechanism of growth inhibition is due to the upregulation of cleaved caspase-3 (**Figures 5A, C**). The tumor derived organoids were compared with untreated control (**Figures 5B, D**). Additional experiments demonstrated SOX10 and SOX2 (stem cell markers) expression in tumor organoids indicated may have some role in radiation and chemotherapy resistance in pancreatic cancer (**Supplementary Figures S6, S7**).

### Radiation treatment response of PDAC organoids to different doses of RT

**Figure 6:**
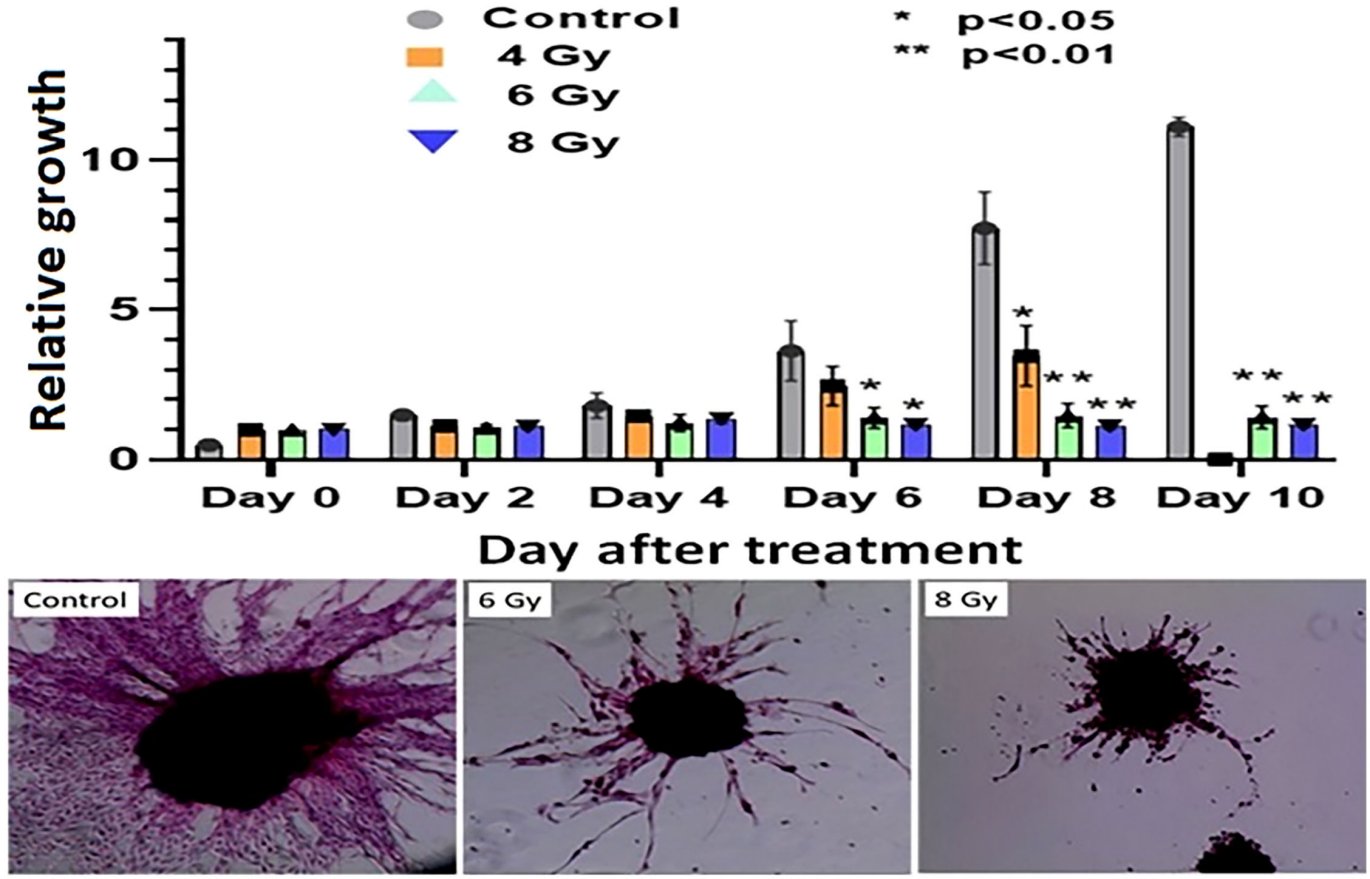
Radiation response of pancreatic tumor organoids (PTO) treated with different doses of RT.Top: Average growth factor defined as organoid size on a specified day divided by organoid size on day 0. Organoids were irradiated with 0 (control), 4, 6, and 8 Gy of radiation over 10 days post treatment. Bottom panel: H&E-stained images of control, 6, and 8 Gy treated organoids on day 10, illustrating sharp decrease in organoid outgrowth after irradiation. Error bars represents 1s standard deviation about the average value.

The response of organoids to different doses of radiation was recorded as the change in average organoid growth factor following radiation treatment. The dense central cores of the 4-Gy RT treated organoids showed reduced growth up to day 8 compared to the untreated control organoids. A statistical significance was found after 6 days of treatment and the organoid growth was inhibited for the full 10 days of observation following treatment. Furthermore, 6- and 8-Gy of RT treatment demonstrated limited growth at 0-6 days incubation (**Figure 6**; **Supplementary Figure S2, S3**). In fact, 8-Gy of fractionated RT exhibited a decrease in area (GF < 1) after 6 days post treatment. Both 6- and 8-Gy RT treated organoids showed a statistically significant (*P* < 0.05) reduction in growth factor (compared to untreated control), and the growth inhibition was highly significant (*P* < 0.01) after 8 days post treatment. As illustrated by H & E staining, organoid growth was noticeably inhibited 10 days post treatment. The fibrotic cellular outgrowth of the tumor organoids was greatly reduced compared to control organoids following 6 and 8 Gy of fractionated RT (**Figure 6**).

### Impact of 3BP anticancer drug alone, and in combination with RT on pancreatic tumor organoid growth

**Figure 7:**
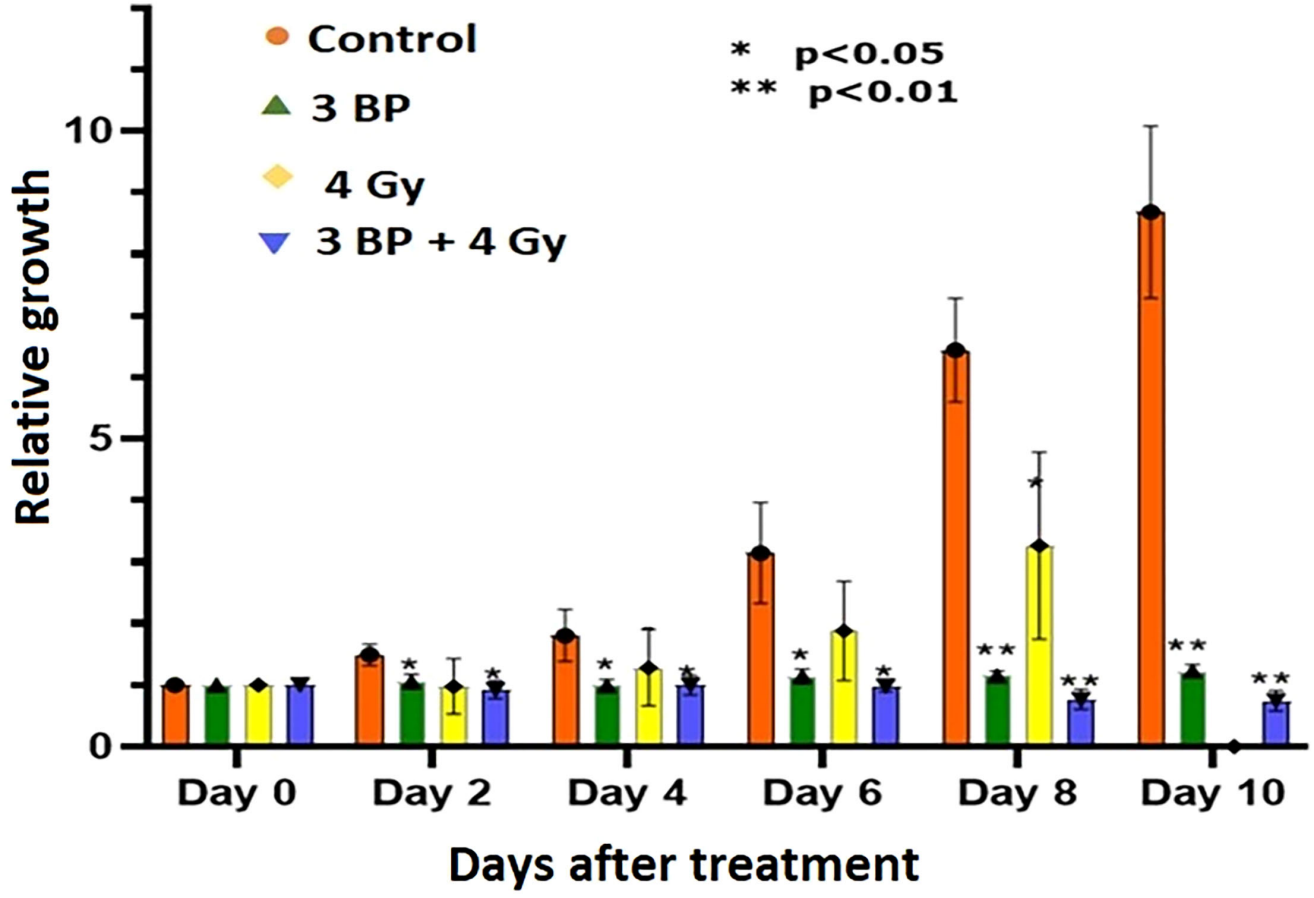
Combined treatment response of pancreatic tumor organoids treated with 3-BP+ radiation.Untreated control (pink bars), 4 Gy radiation treated (yellow bars), 3-BP drug treated (Green triangle), and combined 4 Gy + 3-BP treated (blue triangle) over 10 days post treatment. Error bars represent 1s standard deviation about the average value. Average growth rate factor, defined as the organoid size on a specified day divided by the size on day 0.

Tumor organoids were treated with either 4 Gy RT, 3BP, or a combination of 4 Gy RT + 3BP to monitor the impact of different treatment response (**Figure 6** **;** **Supplementary Figure S4, S5**). The results demonstrated 100 µM of 3BP treatment (sublethal dose) significantly inhibited the dense organoid cores growth (**Supplementary Figure S4**), as compared to control organoids. Notably, 4 Gy of radiation treatment significantly reduced organoid growth factor compared to controls after 8 days. Interestingly, when tumor organoids were treated with a combination of 100 µM of 3BP with 4 Gy of radiation, the growth factor of the organoids was significantly inhibited with an overall GF < 1.0 after 10 days (**Figure 7**).

There were significant noticeable differences in growth inhibition in 3BP only treated, and combined 3BP + 4 Gy RT treated organoids, and differences in growth inhibition in both treatment modalities were statistically significant after 2 days post treatment, and highly significant after 8 days post treatment. Therefore, it is envisaged to conclude that combined treatment of 3BP and radiation was more effective in killing tumor organoids than the individual treatment for the tumors grown for this investigation.

## Discussion

The 3D tumor organoids used in present investigation, were derived from subcutaneous mouse tumor might be relevant for studying patient response to RT or chemo-RT. The current study demonstrated that organoids derived from the mouse tumor tissues recapitulate drug and radiation response similar to *in vivo tumor* (41, 47, 48). Therefore, this study provides an initial indication of the potential use of PDAC tumor organoids as a model to study and predict patient response to treatment (49). In this study, tumor organoids exhibited growth rate characteristics similar to those of untreated and RT-treated *in vivo* tumors. H&E staining and imaging of tumor organoids and corresponding tumor tissues confirmed that organoids also showed the presence of dense fibrotic structures resembling to *in vivo* tumors (50). In addition, treated tumor organoids showed the presence of the same apoptosis biomarkers (cleaved caspase 3) as treated *in vivo* tumors, indicating that the organoids responded to treatment in a similar manner. The corollary studies on patient-derived tumor organoids which also reported activation of cleaved caspase 3 in chemotherapy (paclitaxel and carboplatin) treated tumor organoids and *in vivo* tumors (51, 52).

This study provided an initial indication that PDAC tumor organoids exhibit cellular microenvironments similar to those of the *in vivo* tumors from which they were derived and that organoid response to treatment is similar to that of *in vivo* tumors (43). We believe these initial similarities justify further research into producing and confirming the presence of other therapeutically important components of the pancreatic tumor microenvironment, such as collagen, hyaluronic acid, endothelial cells, and immune cells, and to study the importance of these components to PDAC response to RT, chemotherapy, and Immunotherapy (47, 48). The presence of SOX2 and SOX10 cancer stem cell markers in pancreatic tumor derived organoids delineates role of cancer stem cells in therapy resistance in pancreatic cancer (**Supplementary Figure S6, S74**) (53). Continued work into the engineering and characterization of the full microenvironment of the organoids will help to produce a model that accurately predicts the response of the organoids derived from an individual tumor (e.g., from patient biopsy samples) as a means of predicting a patient’s response to available treatment regimens prior to treatment. Modeling of the full tumor microenvironment would provide a wide range of tumor features in the preclinical organoid model that can be studied in the development of future pancreatic cancer therapies.

This study, to the best of our knowledge, represents the first investigation of differential responses of PDAC organoids to fractionated RT, and combination of radiation with 3BP antitumor drug. Our results demonstrate the potential for using PDAC-derived organoids to study the sensitivity of an *in vivo* tumor to a wide range of treatment regimens such as fractionated RT, chemotherapy, and combined chemo-RT. The dose response study showed that tumor organoids could tolerate 4 Gy of fractionated RT and exhibited only slightly reduced growth compared to untreated controls (**Figure 7**). Further, after treatment with 6 and 8 Gy RT, the tumor organoids initially grew very slowly, with growth completely inhibited beyond day 6 following treatment. Interestingly, 8 Gy of fractionated RT was also effective in completely inhibiting *in vivo* tumor growth in animals, both in our study and as reported by Mahmood et al. (45). We also studied the differential treatment responses of PDAC organoids to 3BP antitumor drug and a combination of 3BP and 4 Gy RT. The combination treatment of 100 µM of 3BP with 4 Gy RT produced nearly the same reduction in tumor organoid growth after 4 days as treatment of the organoids with 6 or 8 Gy RT alone and showed a near-complete inhibition of growth up to day 10.

The 3D tumor organoid has shown promise as an *in vitro* model to predict treatment response to RT and chemotherapeutic drug in an efficient and cost-effective process. Pancreatic tumor tissues and PBMC from a patient could be used to produce 3D pancreatic organoids with microenvironments similar to those of the patient’s tumor and used to test the response to a wide range of available radiation, chemotherapy, immunotherapy, and combination therapies (54, 55). This could provide a method to determine the most effective therapies on an individual patient basis. In present investigation, we have demonstrated the applicability of pancreatic tumor derived organoid to predict radiation and drug treatment response, which is analogous to *in vivo* tumor response. However, this study also has some limitations, mainly simulating the same tumor microenvironment and dense fibrotic stroma present *in vivo*.

## Data availability statement

The original contributions presented in the study are included in the article/**Supplementary Material**. Further inquiries can be directed to the corresponding author.

## Author contributions

HS and JP: Conceptualization, review, editing, writing original draft, supervision. WR, NL and AB: Conceptualization, review, editing. TD, SR, BB, AG: Formal analysis, data curation, performed the experiments, analyzed the data. YP: Biostatistics. JM and WR: Conceptualization. All authors contributed to the article and approved the submitted version.
