# Supplementary material for: Pancreatic cancer derived 3D organoids as a clinical tool to evaluate the treatment response
Source: Front Oncol. 2023 Jan 12;12:1072774. doi: 10.3389/fonc.2022.1072774 (PMC9879007; doi:10.3389/fonc.2022.1072774)
Supplement: Supplementary file 1 [file DataSheet_1.pdf]

SUPPLEMENTARY MATERIAL

The Supplementary Material for this article

Pancreatic Cancer Derived 3D Organoids as a Clinical Tool to Evaluate the Treatment Response.

In Vivo subcutaneous tumor growth procedure.

Panc2 cells were cultured in T175 flasks, and, when growth reached subconfluence, cells were washed with ice-cold 1× phosphate-buffered saline (PBS) twice, trypsinized, and counted in a cell counter (Bio-Rad Laboratories; Hercules, CA). A total of 1×10^6 cells were resuspended in 50 □L cold 1× PBS in prechilled 1.5-mL Eppendorf tubes placed on ice. Before injection animals were anesthetized with isoflurane (3.5%–4 %). Immediately afterward, 1×10^6 cells were mixed with ice-cold Matrigel matrix (Corning Life Sciences; Corning, NY) in a 1:1 ratio (50 + 50 µL). The resulting liquid was then drawn in 0.5-mL syringes fitted with 27-gauge .5-in needles and injected subcutaneously in the right flank of 28 mice.

Organoid production procedure.

Tumors were excised aseptically, transferred in ice-cold Gibco Dulbecco's Modified Eagle Medium (DMEM) F12 (Thermo Fisher Scientific; Waltham, MA) in 145 × 20–mm dishes (Cellstar; Greiner Bio-One North America, Inc.; Monroe, NC), and processed for organoid culture. Excised tumor tissues were cut in 3–4-mm pieces and washed with cold F12 media. Tissue was further chopped with a sterile blade and processed for tissue digestion using a tissue dissociation cocktail (STEMCELL Technologies; Vancouver, CA). Finely chopped tumor tissues in F12 media were transferred to 15-mL tubes and allowed to settle for 5 minutes. The supernatant was removed and mixed with 5 mL of tissue dissociation cocktail, incubated at 37°C for 20 minutes. Using a 10-mL pipette, the tissue was vigorously mixed by up-and-down pipetting (7–8 times), and the tissue was left to settle. The supernatant was removed, and the digestion step was repeated. The supernatant was transferred to 50-mL tubes with a 70 µm cell strainer (STEMCELL Technologies; Vancouver, CA) on top, and the flow-through was discarded. The strainer was reversed on 50-mL ice-cold tube, and 5 mL DMEM F12 media were added to the strainer to recover the tissue fragments. Tubes were centrifuged at 400×g for 5 minutes, and the supernatant was removed leaving only the tissue pellet. The tissue pellet was placed on ice and mixed with ice-cold Matrigel (100 µl) using a prechilled (–20°C) 200-µl pipette tip. The pellet was resuspended in Matrigel by pipetting up and down 10 times, taking care to avoid introducing bubbles.

Fig. S1:

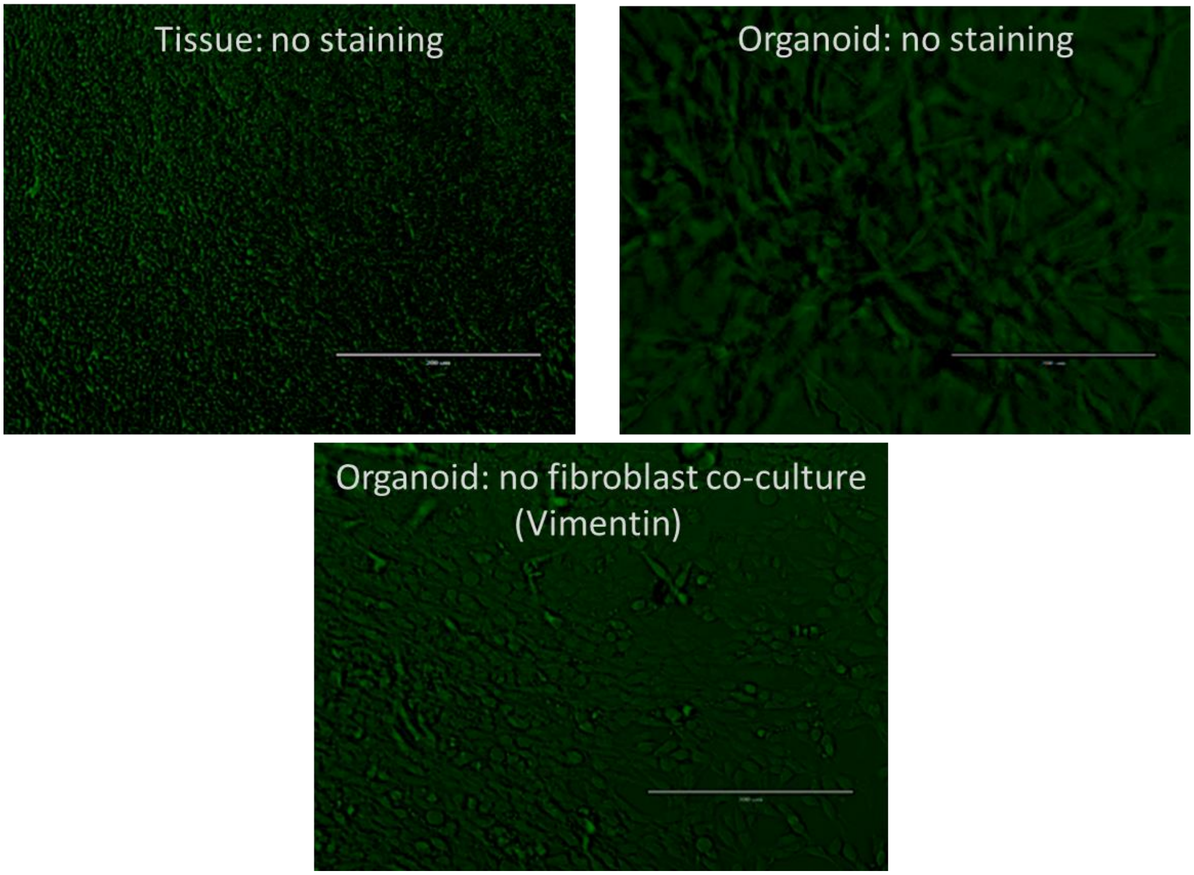

Figure S1: Control images for IHC imaging of excised tumor tissues and organoids. Shown are IHC images of tumor tissues and organoids with no staining for Vimentin showing no luminescence signal in the absence of staining. Also shown are IHC images of organoids that were not co-cultured with fibroblasts or stem cells showing that no expression of Vimentin is present.

Fig. S2

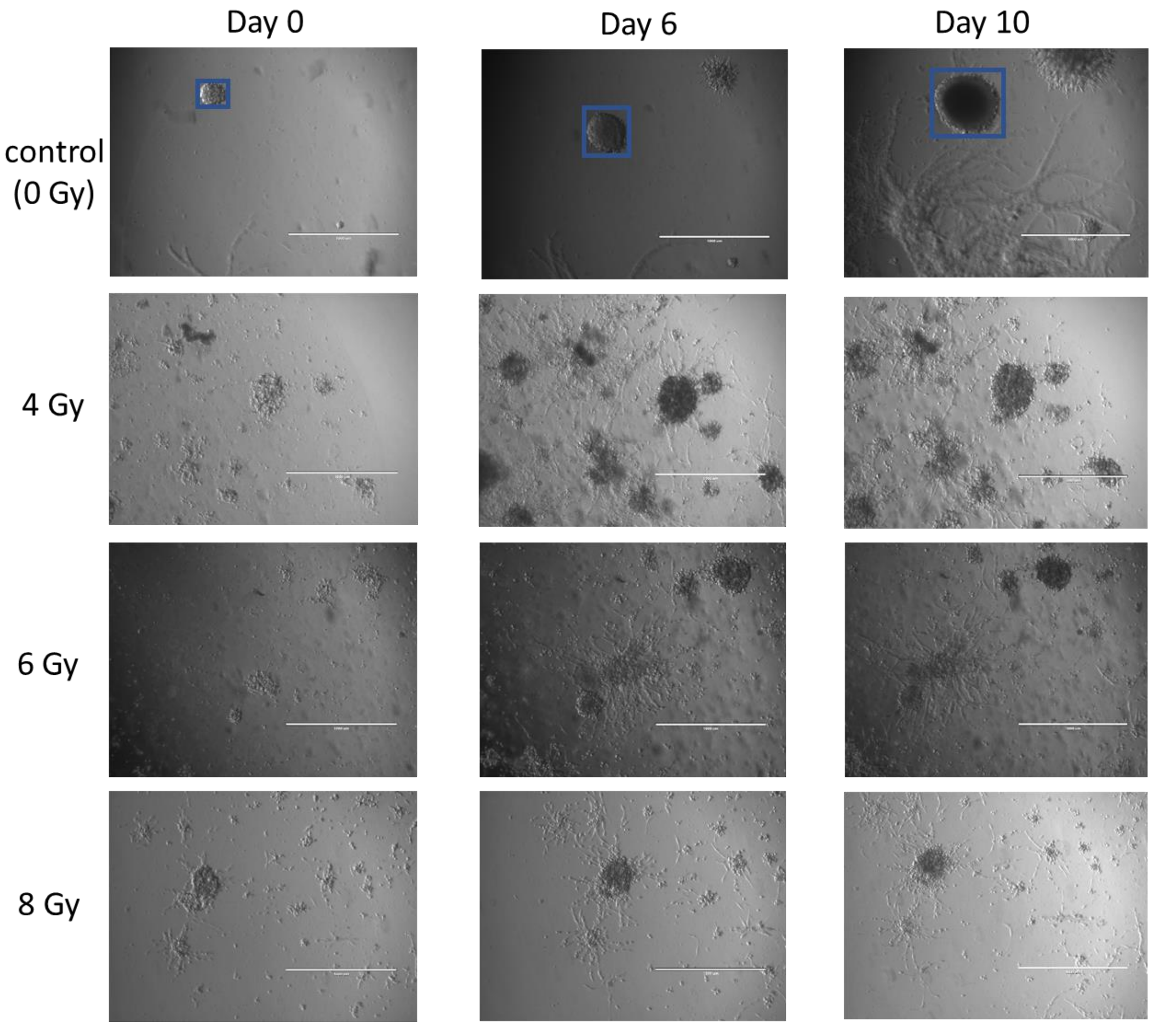

Figure S2: Images of organoids treated with radiation therapy on days 0 (day of treatment), 6 and 10 after treatment. Blue rectangles around organoids demonstrate how the size of the organoid was determined. The organoid size was calculated as (length x width) of the blue rectangle. Scale bar = 1000 µm.

Fig. S3:

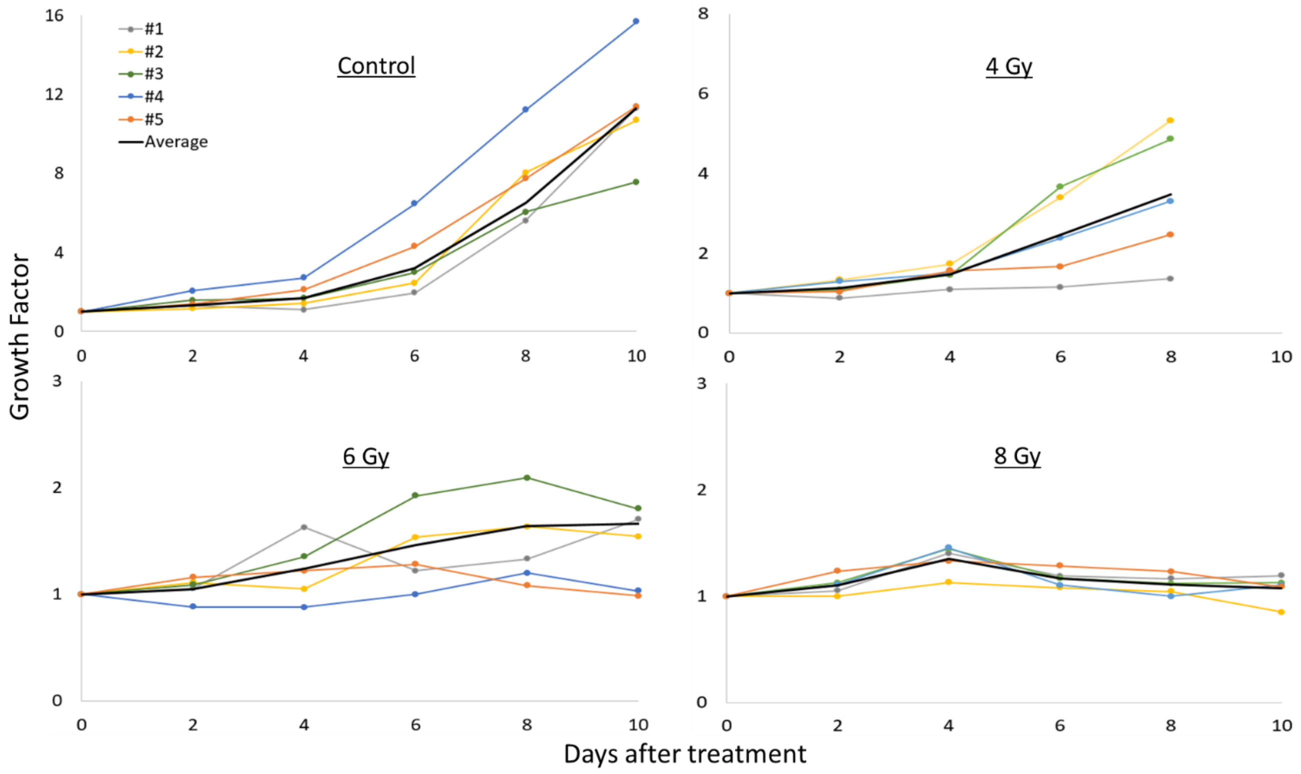

Figure S3: Individual and average growth of five organoids (used to create Figure 4) for ten days following treatment with 0 Gy (Control), 4 Gy, 6 Gy, and 8 Gy of radiation.

Fig. S4:

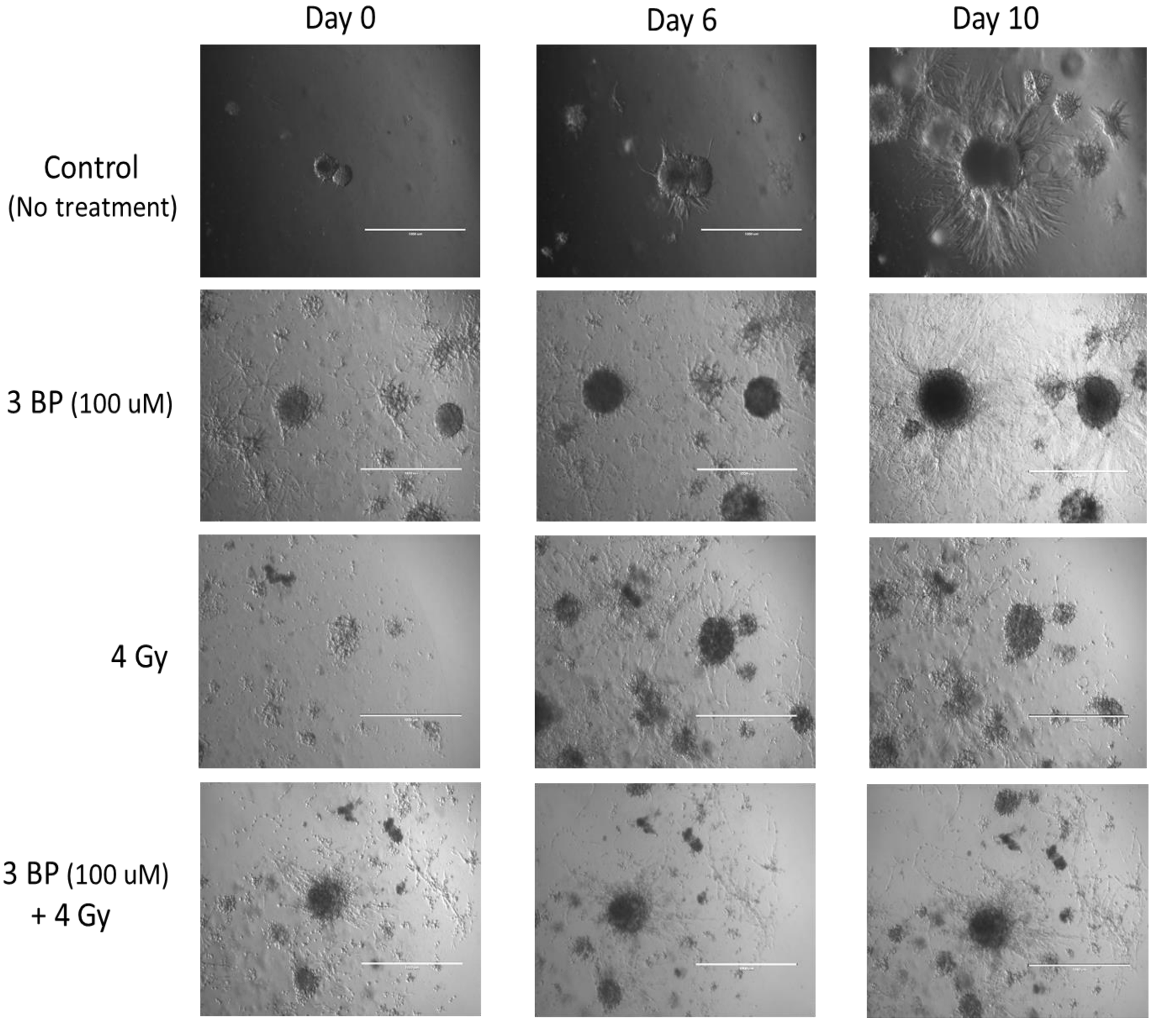

Figure S4: Images of organoids treated with 3BP, 4 Gy radiation therapy or combined 3 BP + 4 Gy radiation therapy on days 0 (day of treatment), 6 and 10 after treatment. Scale bar = 1000 µm.

Fig S5:

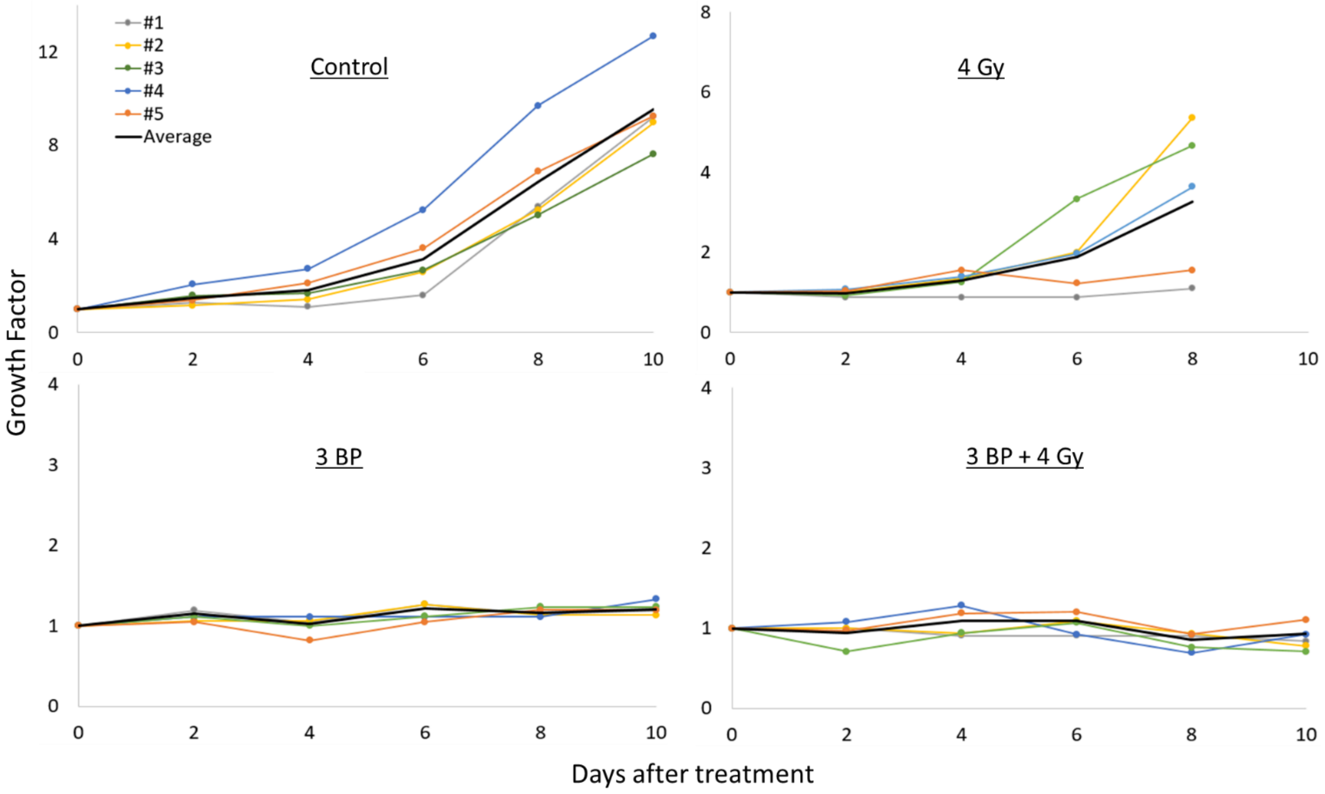

Figure S5: Individual and average growth of five organoids (used to create Figure 5) for ten days following no treatment (Control) and treatment with 4 Gy of radiation therapy, 3BP, and combined 3BP + 4 Gy radiation therapy.

Figure S6:

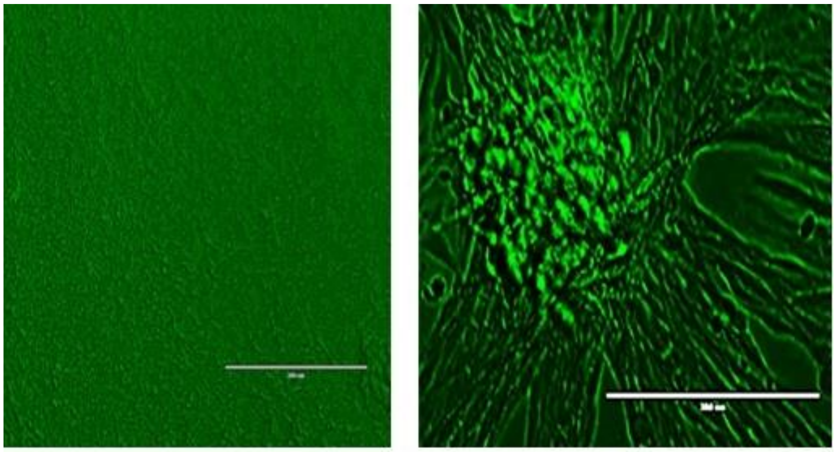

A B

Fig. S6: IHC staining of SOX10 in pancreatic tumor organoids showing A, negative control ; B, positive staining of SOX 10.

Fig. S7:

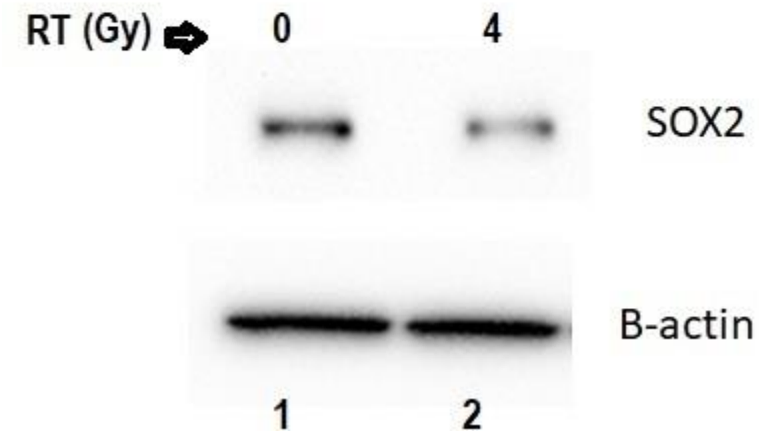

Fig. S7: Identification of SOX2 stem cell marker in pancreatic tumor organoids treated with lane 1, 0 Gy; and lane 2, 4 Gy of radiation.

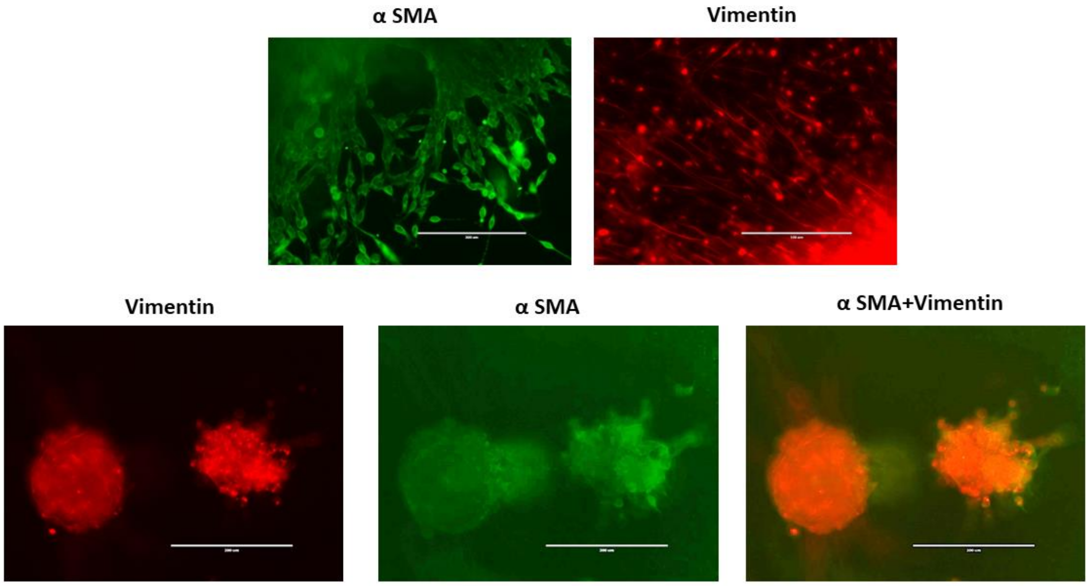

Suppl Fig. S8: Single and co-staining of pancreatic tumor organoids using vimentin and αSMA antibodies.
